# The comfort assessment in healthy adults during constant‐flow mode in noninvasive ventilator

**DOI:** 10.1111/crj.13459

**Published:** 2021-12-06

**Authors:** Juanjuan Yao, Wei Li, Mengmeng Peng, Kaixin He, Dedong Ma, Hongxiu Lu

**Affiliations:** ^1^ Institute of Respiratory Monitoring and Support Shandong University Jinan China; ^2^ Biomedical Engineering Institute, School of Control Science and Engineering Shandong University Jinan China; ^3^ Clinical Medicine School Shandong University Jinan China; ^4^ Department of Pulmonary and Critical Care Medicine, Qilu Hospital Shandong University Jinan China; ^5^ Department of Anesthesiology Affiliated Hospital of Shandong University of Traditional Chinese Medicine Jinan China

**Keywords:** comfort, constant‐flow mode in noninvasive ventilator, high‐flow nasal cannula, noise level

## Abstract

**Background:**

From the point of view of machine construction and hydrodynamics, this paper innovatively proposes that the essence of high‐flow nasal cannula (HFNC) is a constant‐flow mode in noninvasive ventilator (NIVCFM). This study enrolled healthy adults as study subjects to assess the subjective comfort assessed by visual analog scoring scale of NIVCFM/HFNC and objective comfort measured by the noise level generated by NIVCFM/HFNC, aiming to provide a scientific basis for the rational clinical application of NIVCFM/HFNC.

**Methods:**

Forty‐four healthy adults participated in this study. The noise generated by NIVCFM/HFNC is measured, and the comfort is evaluated during NIVCFM delivery at flow rates of 0, 5, 10, 15, 20, 25, 30, 35, 40, 45, 50, 55, and 60 L/min.

**Results:**

When the ventilator flow rate is 60 L/min, the maximum noise is 65.9 dB, increasing noise by 23.7 dB from a baseline of 42.2 dB at the flow rate of 0 L/min. There was a strong nonlinear positive correlation between the noise level and the flow rates. The median score for dry mouth, nose or throat, dysphagia, sore throat, and other discomfort was 0. The median score for dyspnea was 0 at 0–30 L/min, 1 at 35–55 L/min, and 2 at 60 L/min.

**Conclusions:**

The grater the flow rate, the greater the noise generated by NIVCFM/HFNC (<65.9 dB). The maximum flow rate that most healthy adults can able to tolerate is 30 L/min, and the main discomfort is dyspnea.

## 1 INTRODUCTION

High‐flow nasal cannula (HFNC) is a noninvasive respiratory support technique, which can provide accurate oxygen concentration (21%–100%) and up to 60 L/min of gas flow rate through air oxygen mixing device. The delivered gas mixture is actively heated up to 37°C and humidified to 100% relative humidity via a heated humidifier connected to the special nasal cannula through a single‐limb noncondensing insulated circuit. From the point of view of machine construction, the nasal cannula of HFNC is unsealed, and its aperture is smaller than that of the outer nostrils of the human body, so HFNC allows a certain amount of air leakage when providing respiratory support. From the perspective of hydrodynamics, the human nasal cavity can be seen as a channel with a fixed aperture, and the high gas flow generated by the HFNC may have a resistance to the human exhaled air flow. The higher the flow rate of the gas, the greater the pressure generated. That is, the HFNC produces a constant‐flow with changing pressure. In general, the essence of HFNC is a constant‐flow noninvasive support system with high allowable leakage. In other words, HFNC is a constant‐flow mode in noninvasive ventilator (NIVCFM).

More and more people pay attention to the noise exceeding the standard in hospitals. Most of the existing studies^1^ only briefly talk about the noise generated by the NIVCFM/HFNC, although there is little research on how much noise can be produced by NIVCFM/HFNC at different flows (0–60 L/min). Hugo Lenglet et al.^2^ placed the sound meter 1 m from the HFNC machine in the emergency room found that HFNC generated 55 dB of noise at 40 L/min. Kubo et al.^3^ measured the noise at the distance HFNC (30, 40, 50, and 60 L/min) machine 1 m using a digital sound measuring instrument in the absence of human use and found that the noise would increase with the increase of the flow rate.

Patients receiving low‐flow oxygen therapy have mild discomfort or no discomfort during treatment, so clinical guidelines do not recommend routine use of humidifiers for low‐flow oxygen therapy.^4^ However, critically ill patients with acute respiratory failure are usually treated with high‐flow oxygen through a mask. Breathing dry and cold oxygen can provoke dryness of the mouth, nose, throat, and respiratory tract, which may frequently result in discomfort and pain.^5^ A study^6^ has reported that comparing with nasal catheter oxygen inhalation, mask oxygen inhalation, venturi mask oxygen inhalation, and noninvasive ventilation, the overall comfort (including interface comfort, airway dryness, dysphagia and sore throat, etc.) of the patients with HFNC application is higher and more tolerable.

There are studies using a digital scoring scale or visual analog scoring scale to assess subjective HFNC comfort. Cuquemelle et al.^1^ compared comfort in receiving nonhumidified general oxygen therapy (9 L/min) and HFNC (12 L/min). The results showed that HFNC treatment had lower oral, nose, and throat dryness scores than normal oxygen treatment. Tommaso Mauri et al.^7^ compared the comfort level of HFNC at different temperatures. They found that the comfort assessed by the patient's visual digital scale was higher at 31°C; on the contrary, comfort was not affected by the flow rate (30 L/min, 60 L/min).

Our study enrolled healthy adults as study subjects to assess the subjective comfort assessed by visual analog scoring scale of NIVCFM/HFNC and objective comfort measured by the noise level generated by NIVCFM/HFNC, aiming to provide a scientific basis for the rational clinical application of NIVCFM/HFNC.

## 2 METHODS

### 2.1 Subjects

Healthy adults were consecutively recruited and considered eligible if they were between the ages of 18 and 30 years old and had no upper respiratory tract anatomic malformations, neuromuscular disease, chest disease, and respiratory disease. Not only that, but also excluded adults with a history of smoking and those who take drugs that influence cardiopulmonary function. All of the participants accepted the research voluntarily and signed the written informed consent form.

### 2.2 Study procedures

Age, sex, and body mass index (BMI) were recorded. A brief introductory session was held to familiarize all subjects with the interventions they would be receiving. Before the measurements, all subjects maintained natural breathing for 10–15 min to reach a physiological steady state. Afterwards, they were treated by constant‐flow mode in noninvasive ventilator. Institutional review committees approved the study, and written informed consent was obtained from healthy adults.

### 2.3 Application of constant‐flow mode in noninvasive ventilator

The subjects were in a vertical sitting position during the test and breathed through their nose with their mouth closed while wearing the HFNC (Veoflo®, Flexicare Medical Ltd, UK), which is made of soft silicon, with a wider bore (internal diameter = 4 mm) than traditional nasal cannula, and is designed specifically to deliver heated and humidified gas. Conditioned room air (21% O_2_, 0.04% CO_2_) was delivered by the constant‐flow mode in non‐invasive ventilator (GA ST40P, CURATIVE, Beijing, China) at flow rates of 0, 5, 10, 15, 20, 25, 30, 35, 40, 45, 50, 55, and 60 L/min. The heated humidifier (VHC 20, INSPIRED MEDICAL, Guangdong, China) temperature was set to 37°C, and relative humidity was close to 100%. Before changing the flow rate, the volunteers were asked to take off the nasal cannula and wear it again after 2 min.

### 2.4 Objective comfort: Noise measurements

For the noise measurements, we used the sound level meter (8922, AZ® Instrument Corp., Taiwan China). We measured the noise levels for NIVCFM/HFNC at a distance of 1 m with the flows of 0, 5, 10, 15, 20, 25, 30, 35, 40, 45, 50, 55, and 60 L/min. Noise data were continuously recorded and later analyzed on a personal computer using Handheld Meter's Data Logger V3.10 (Kingst History Data Review, Beijing, China). Recordings were started after a period of stabilization, ensuring at least five breaths of stable noise data. Mean noise level was calculated by averaging the noise of the 1‐min recording.

### 2.5 Subjective comfort score: Visual analog scoring scale

**FIGURE 1:**
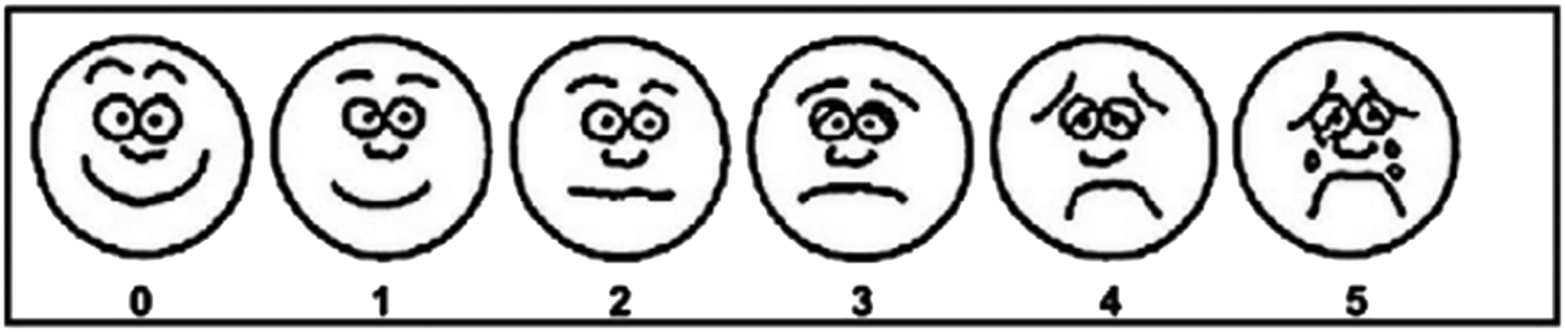
Comfort score. A score of 0 indicates no discomfort; 1, little bit discomfort; 2, little more discomfort; 3, even more discomfort; 4, whole lot more discomfort; 5, worst discomfort

Comfort was assessed by asking health adults to rate comfort related to the symptoms of airways dryness (mouth, nose, or throat dryness; dysphagia; expiratory dyspnea; throat pain; and other discomfort) by a visual analog scale (Figure 1).^8^

### 2.6 Statistical analysis

Data from the sound level meter were downloaded into Excel (Microsoft, Redmond, Washington) spreadsheets. The Excel data were then function‐fitted using MATLAB 7.11's (MathWorks, Natick, Massachusetts) Curve Fitting Toolbox with the use of the least square method. The data were analyzed using the statistical package for social sciences 22.0 (IBM® SPSS Statistic's®, Chicago, IL, USA) for Windows. The categorical variables were expressed as frequencies and the continuous variables as the mean ± *SD* when the data followed a normal distribution. For the comparison between noises at different flow rates, the single‐factor analysis of variance (ANOVA) test or Kruskal–Wallis nonparametric test was used. Statistical significance was established at *P* < 0.05.

## 3 RESULTS

Twenty‐three male and 21 female participated in this study with a mean age of 23.1 ± 2.1 years old and a BMI of 21.9 ± 3.7 kg/m^2^.

### 3.1 Noise generated by NIVCFM/HFNC

Constant‐flow mode in noninvasive ventilator increased noise by 23.7 dB at 60 L/min from a baseline of 42.2 dB at the flow rate of 0 L/min (Table S1).

**FIGURE 2:**
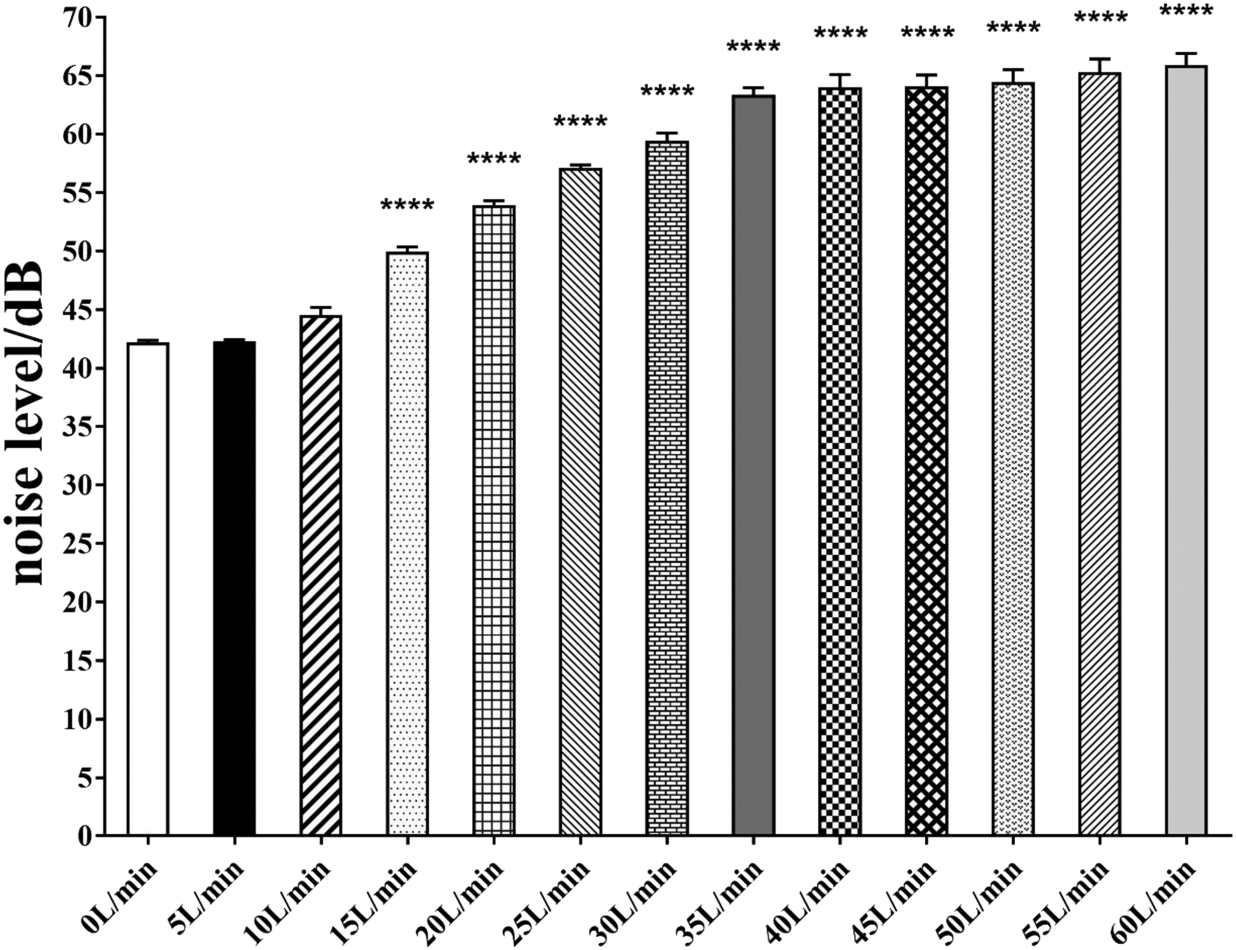
Effect of flow rates on noise level. All *P* values versus the flow rate of 0 L/min. Data are mean ± standard error. ****P < 0.0001

Comparing with 0 L/min, constant‐flow mode in noninvasive ventilator induced a significant increase in noise after 15 L/min (*P* < 0.0001), although there was a slow increase after the flow rate was larger than 35 L/min (Figure 2).

MATLAB 7.11 was able to construct two‐order Fourier functions to fit noise curves (Table S2). There is a strong nonlinear positive correlation between noise and delivered gas flow rate (*R* ^2^ = 0.87) (Figure S1).

### 3.2 Comfort score

**FIGURE 3:**
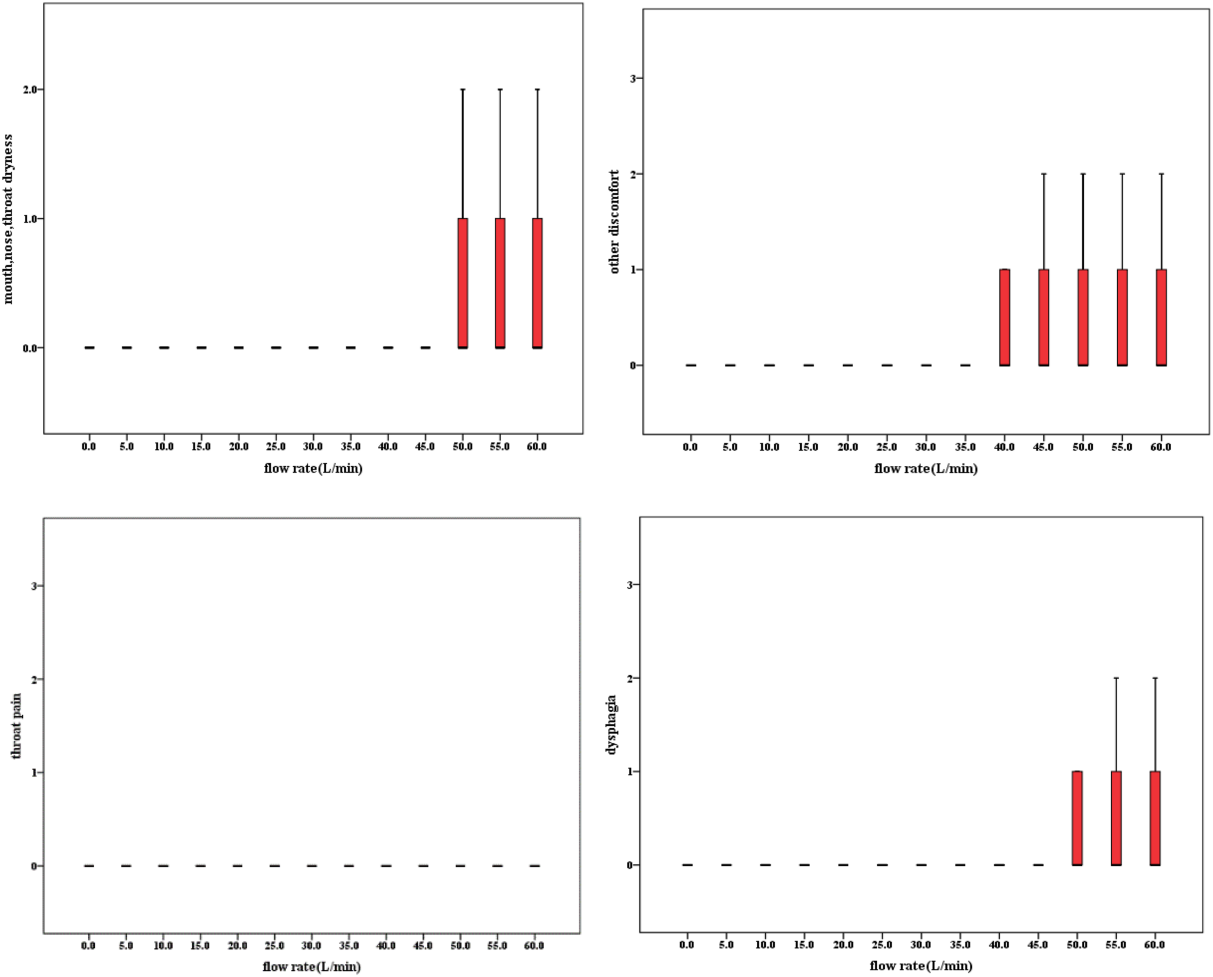
Box and whisker charts of comfort scores for mouth, nose, or throat, dryness, dysphagia, throat pain, and other discomfort at different flow rates

**FIGURE 4:**
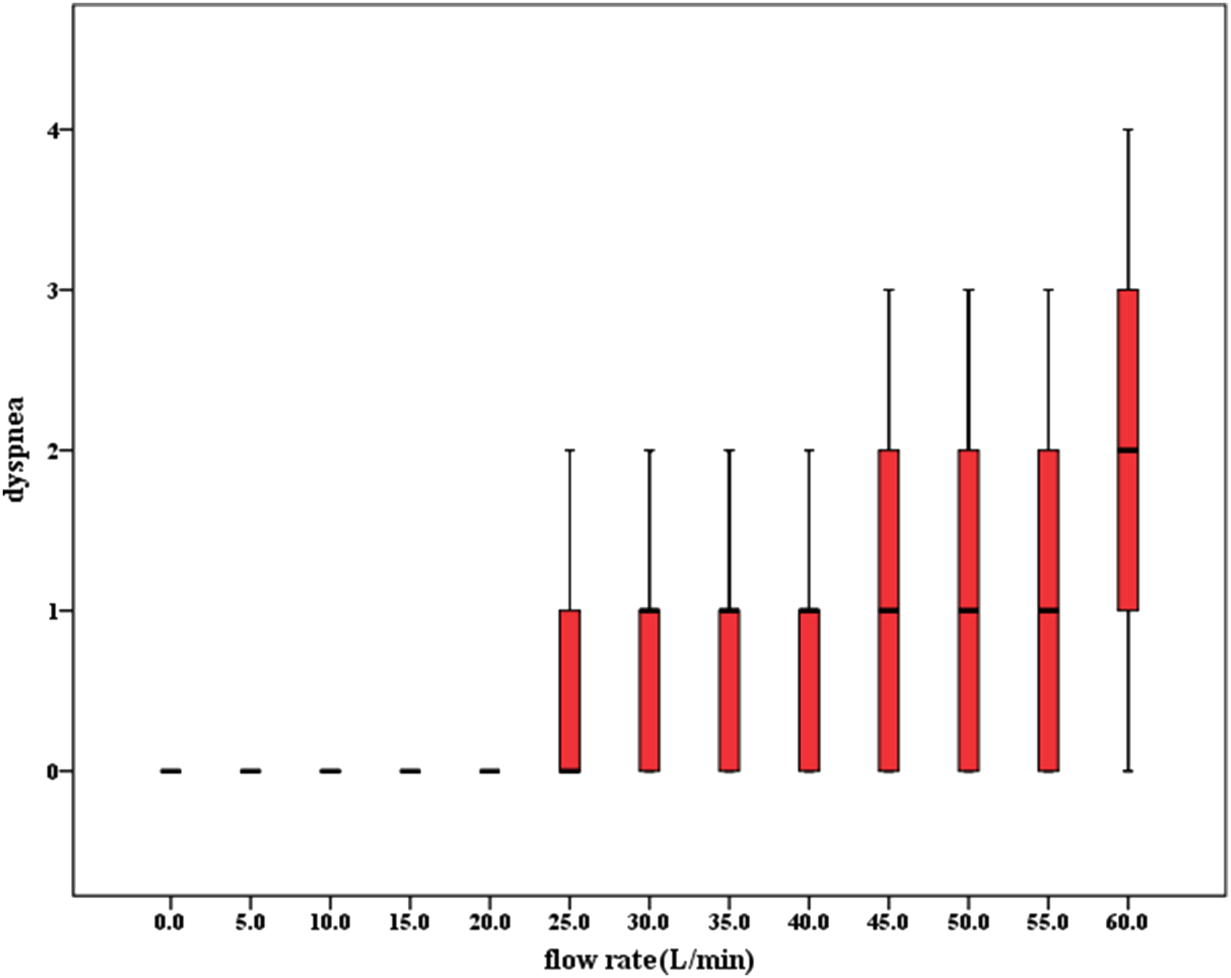
Box and whisker charts of comfort scores for dyspnea at different flow rates

The median score of mouth, nose, or throat dryness score, dysphagia score, throat pain score, and other discomfort score showed in Box, and whisker plots were all 0 at different ventilator flow rates (Figure 3). The median score for expiratory dyspnea was 0 at 0–30 L /min,1 at 35–55 L/min, and 2 at 60 L/min (Figure 4).

When the flow rate >45 L/min, nine subjects felt slight discomfort due to mouth, nose, or throat dryness and dysphagia (1 point); four subjects felt a little discomfort due to mouth, nose or throat dryness and dysphagia (2 points); two subjects complained of sore throat; and throat pain was considered obvious at 55 and 60 L/min (3 points).

Most subjects had different degrees of other discomfort at 30 L/min (1–3 points), such as tympanic flatulence, nasal wing and nasal septum acid distension during exhalation, and sinus pain, wanting to swallow saliva (but with resistance). When the flow rate is >35 L/min, one subject complained that he had a transient dizziness when he had just put on and removed the nasal obstruction catheter. When the flow rate is >45 L/min, one subject complained of slight swelling of the inner canthus; one subject complained of gas entering the stomach when swallowing. When the flow is >50 L/min, one subject complained of mild nausea. When the flow rate increased from 30–40 to 40–50 L/min, the discomfort of individual subjects was alleviated, but the discomfort was aggravated when the flow rate was 50–60 L/min.

## 4 DISCUSSION

### 4.1 Effect of flow rate on noise level

According to some studies,^3^, ^9^, ^10^ the NIVCFM/HFNC is the main determinant of noise. A strong nonlinear positive correlation between noise and flow rate is also demonstrated in this study (adjusted *R* ^2^ = 0.87). That is, the noise will increase with the increase of ventilator flow. This study also found that when the ventilator flow rate is 60 L/min, the noise is the largest (65.9 ± 4.7 dB). The noise generated by the NIVCFM/HFNC at 15–60 L/min is statistically significant compared with that generated by NIVCFM/HFNC at 0 L/min (*P* < 0.05). When the flow rate is ≥40 L/min, the change of noise with the flow rate is not obvious.^11^ A study has shown that noise levels that stimulate the adrenal cortex axis with a noise threshold of 68 and 70 dB can have an impact on the cardiovascular system, such as peripheral vasoconstriction, increased heart rate, increased blood pressure, and 50–75 dB can cause significant sleep disorders in infants. Although the maximum noise generated in this study is 65.9 ± 4.7 dB, because there are other noise sources in the hospital, we suggest the use of higher flow rates should be avoided as much as possible in clinic; future NIVCFM/HFNC device design should pay attention to minimize the noise to prevent the harmful effects of excessive exposure to environmental noise on patients. In this study, breathing filters were installed on the intake side to filter moisture, dust, and bacteria; Kubo^3^ found it can also reduce noise. In addition, medical staff can also let patients wear hearing protection devices, such as earplugs. It should be noted that the findings of this study are related to the equipment used, that is, CURATIVE GA ST40P noninvasive ventilator, and cannot be extrapolated to other equipment.

### 4.2 Effect of flow rate on comfort score

Dry and cold gas provided by traditional oxygen therapy can lead to dry upper airway, mucosal inflammation, influence mucociliary clearance, increase airway resistance, and promote atelectasis and ultimately reduce patient comfort and therapeutic effect.^12^ This study found that the median score of mouth, nose or throat dryness score, and throat pain score were all 0 at different flow rate of NIVCFM/HFNC; there is no obvious discomfort in score of mouth, nose, or throat dryness score on average, but some individuals did experience discomfort. This may be because the NIVCFM/HFNC provides heating, humidifying gas consistent with the physiological temperature and humidity of the human airway. Roca et al.,^13^ in a cross‐study of 20 patients with acute respiratory failure mainly caused by pneumonia, found that compared with mask oxygen inhalation using a bubble humidifier, HFNC could improve overall comfort, reduce dyspnea score, and reduce oral dryness. This study found that in terms of dyspnea, subjects were not unwell at 0–30 L/min; subjects felt a little discomfort at 35–55 L/min, showing resistance to exhalation; subjects felt slight discomfort at 60 L/min, showing increased resistance to exhalation; and subjects felt a little discomfort when the flow was greater than 45 L/min, showing occasional need to open mouth to breathe. This is because when the flow rate is greater than the expiratory flow rate, the gas provided by the ventilator will cause certain resistance to the expiratory flow, resulting in difficulty in expiratory. When the flow rate increased from 30–40 to 40–50 L/min, the discomfort of subjects was alleviated, but the discomfort was aggravated when the flow rate was 50–60 L/min. We speculate that this may be because subjects regulate breathing autonomously when using a NIVCFM/HFNC for a period of time; although breathing will become difficult again when the NIVCFM/HFNC flow is too high to inhibit spontaneous breathing. This suggests that medical staff can start from low‐flow rate according to patient's tolerance and then gradually increase to treatment flow rate after patient adapts.

In a study of 32 healthy subjects, the researchers found that when the flow rate was set too high (flow rate > 40 L/min), some subjects complained of shortness of breath, suffocation, and significantly increased swallowing time and dysphagia. During this study, subjects complained of sore throat, bulging tympanic membrane, sore nasal wing and nasal septum, and sinus pain, wanting to swallow saliva (but with resistance), transient head confusion, slight swelling of inner canthus, gas entering stomach when swallowing, slight nausea, and so on. The main reason is that when the flow rate is high, the relative humidity of the gas cannot reach 100%. A study^12^ has found that compared with 40 L/min, the absolute humidity of a MR850 heating humidifier with a MR290 humidifier is lower at 60 L/min. Therefore, when the NIVCFM/HFNC flow rate is higher than 30 L/min, it should be noted that humidification may not be sufficient. This study also concluded that high gas flow would have a certain impact on the tympanic membrane, nasal alar, nasal septum, and inner canthus. Therefore, for patients with ear or eye disease, try not to use NIVCFM/HFNC treatment or use low‐flow NIVCFM/HFNC, to avoid damage to the eyes or ears. During the test, we also found that when the ambient temperature is lower than the 37°C provided by the ventilator, there will be condensate water in the ventilator pipeline, and the high‐speed jet gas would spray the condensate water into the nasal cavity. Therefore, attention should be paid to reducing the temperature difference between the environment and the ventilator conveying gas in clinical application. Furthermore, Chikata et al.^14^ also found that in addition to ambient air temperature, both the ventilator flow rate and the technical design of the ventilator air supply pipeline affect the amount of condensation.

In this study, we found that there are some individual differences in comfort, probably because comfort is affected by psychological factors, and everyone has different tolerance to discomfort and pain. The ex‐post analysis of FLORALI study^15^ showed that poor comfort was the only predictor of tracheal intubation in patients with acute respiratory distress syndrome using NIVCFM/HFNC in 1 h, suggesting that comfort was associated with improved clinical outcome. Studies have shown that improving patient comfort by selecting the minimum flow rate to achieve treatment goals reduces the need for sedation and reduces the risk of delirium.^16^ This suggests that medical staff should pay attention to the minimum flow rate under the premise of ensuring the therapeutic effect, so as to improve the patient's compliance and therapeutic effect.

## 5 CONCLUSIONS

The grater the flow rate, the greater the noise generated by NIVCFM/HFNC (<65.9 dB). The maximum flow rate that most healthy adults can able to tolerate is 30 L/min, and the main discomfort is dyspnea.

## CONFLICT OF INTEREST

The authors have no conflicts of interest to declare.

## AUTHOR CONTRIBUTIONS

Juanjuan Yao performed research; Kaixin He collected the data; Hongxiu Lu performed the data curation and analyzed the data; Mengmeng Peng write the draft; Wei Li performed the editing; Dedong Ma was responsible for the project administration.

## DATA ACCESSIBILITY STATEMENT

The data that support the findings of this study are available from the corresponding author upon reasonable request.

## ETHICAL STATEMENT

The authors are accountable for all aspects of the work in ensuring that questions related to the accuracy or integrity of any part of the work are appropriately investigated and resolved. The study was conducted in accordance with the principles of the Declaration of Helsinki and approved by the Research Ethics Committee of Qilu Hospital. Patient approval and informed consent were waived because the study involved a retrospective review of patient records. All parties signed informed consent, and the project has been approved by the Ethics Committee of Shandong University Qilu Hospital.

## FINANCIAL DISCLOSE

None.

## Supporting information


**Table S1:** Noise generated by Constant‐flow mode of non‐invasive ventilator during nasal high‐flow rates of 0,5,10,15,20,25,30,35,40,45,50,55,60 L/min
**Table S2:** The mean (with 95% confidence bounds) of each coefficient of noise level function
**Figure S1.** The fitting curve of Fourier of noise level

## Data Availability

Data available on request from the authors.
