# Supplementary material for: The comfort assessment in healthy adults during constant‐flow mode in noninvasive ventilator
Source: Clin Respir J. 2021 Dec 6;16(2):123–9. doi: 10.1111/crj.13459 (PMC9060056; doi:10.1111/crj.13459)
Supplement: Supplementary file 1 — Table S1: Noise generated by Constant‐flow mode of non‐invasive ventilator during nasal high‐flow rates of 0,5,10,15,20,25,30,35,40,45,50,55,60 L/min Table S2: The mean (with 95% confidence bounds) of each coefficient of noise level function Figure S1. The fitting curve of Fourier of noise level [file CRJ-16-123-s001.docx]

**S1:** Noise generated by Constant-flow mode of non-invasive ventilator during nasal high flow rates of 0,5,10,15,20,25,30,35,40,45,50,55,60 L/min

| flow rate/L⋅min^-1^ | Noise level/dB |
| --- | --- |
| 0 | 42.2±0.9 |
| 5 | 42.3±0.6 |
| 10 | 44.6±2.8 |
| 15 | 50.0±1.7 |
| 20 | 53.9±1.6 |
| 25 | 57.1±1.2 |
| 30 | 59.4±3.0 |
| 35 | 63.4±2.7 |
| 40 | 64.0±4.8 |
| 45 | 64.1±4.1 |
| 50 | 64.5±4.7 |
| 55 | 65.3±5.0 |
| 60 | 65.9±4.7 |

| **S2:** The mean(with 95% confidence bounds) of each coefficient of noise level function | |
| --- | --- |
| coefficient | mean(with 95% confidence bounds) |
| a_0_ | 56.93(52.59,61.27) |
| a_1_ | -11.59(-13.08,-10.10) |
| a_2_ | -3.46(-6.99,0.08) |
| w | 0.06(0.03,0.08) |
| b_1_ | -1.61(-4.37,1.16) |
| b_2_ | 0.24(-1.81,2.29) |


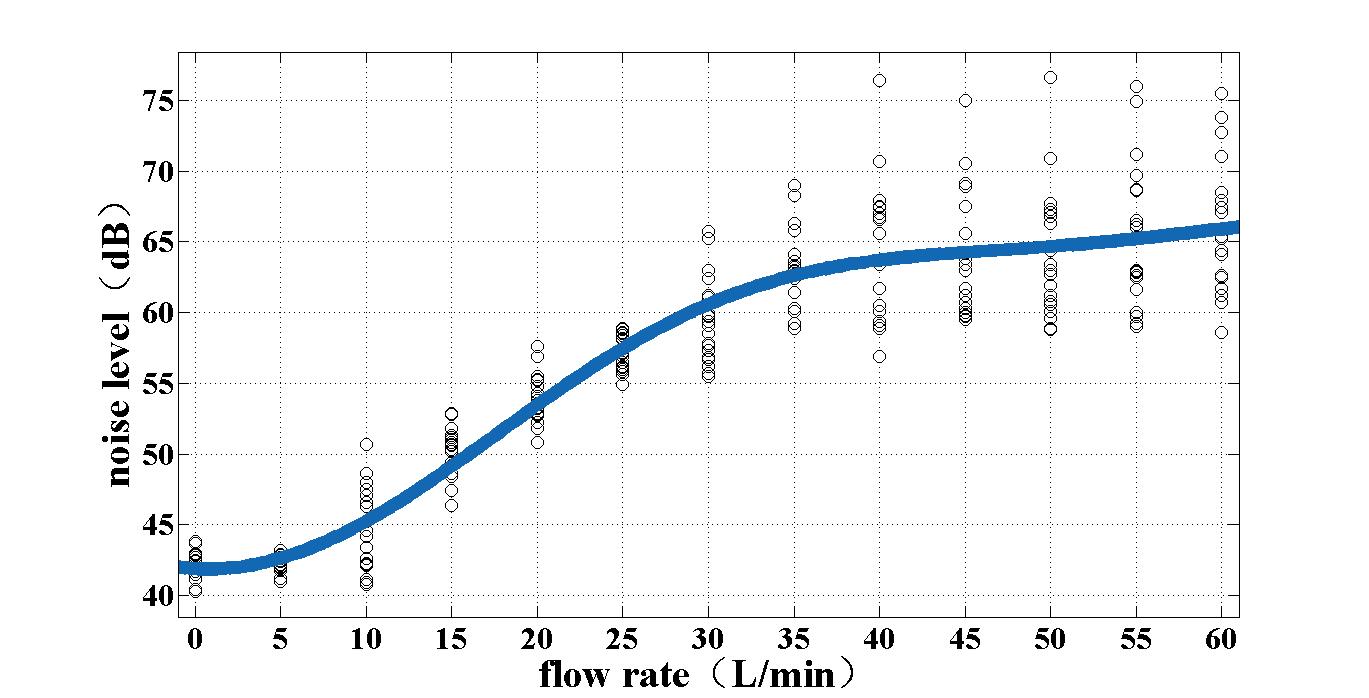


S3. The fitting curve of Fourier of noise level

S4 The Fourier equations

The one-order Fourier function is as follows:

$f\left( x \right)=a_{0}+a_{1}\times\cos\left( x\times w \right)+b_{1}\times\sin\left( x\times w \right)$

The two-order Fourier function is as follows:

$\begin{aligned} f\left( x \right)=a_{0}+a_{1}x\cos\left( xxw \right)+b_{1}\times\sin\left( x\times w \right)+ \\ a_{2}\times\cos\left( 2\times x\times w \right)+b_{2}x\sin\left( 2\times x\times w \right) \end{aligned}$

The three-order Fourier function is as follows:

$\begin{aligned} f\left( x \right)=a_{0}+a_{1}x\cos\left( x\times w \right)+b_{1}\times\sin\left( x\times w \right)+ \\ a_{2}\times\cos\left( 2\times x\times w \right)+b_{2}\times\sin\left( 2\times x\times w \right)+ \\ a_{3}\times\cos\left( 3\times x\times w \right)+b_{3}\times\left( 3\times x\times w \right) \end{aligned}$

In these Fourier functions, x represents nasal high flow rate. f(x) represents noise level. w, a_0_-a_3_, b_1_-b_3_ represent the coefficients of factor x. x=0, 5, 10, 15, 20, 25, 30, 35, 40, 45, 50, 55, 60.
